# Illinois State Dental Society

**Published:** 1888-07

**Authors:** C. N. Johnson


### ILLINOIS STATE DENTAL SOCIETY.

### TWENTY-FOURTH ANNUAL MEETING.

REPORTED FOR THE INDEPENDENT PRACTITIONER.

BY C. N. JOHNSON, L. D. S., D. D. S.

CONTINUED FROM PAGE 319.

### WEDNESDAY EVENING.

A special order for the evening was the description, by Dr. C. C. Carroll, Meadville, Pa., of his method of casting aluminum plates. Briefly, it was as follows: The principal objections, heretofore, to the use of aluminum for artificial dentures were that it could not be successfully soldered, and that when used in connection with rubber it would disintegrate. Efforts were then made to cast aluminum, but a difficulty arose in the fact that it would not pour well into interstices, and thus failed to make a sharp cast. This was overcome by the application of pneumatic pressure, so exerted as to force it into the impression; but then it was found that it would contract. These difficulties have all been remedied. The disintegration was due to impurities (principally of iron), as aluminum has a great affinity for other metals, and was overcome by care in purifying. The contraction was rectified by alloying it with some metal which expands at the same time that the aluminum contracts. The metals now used in combination with aluminum to obtain the best results in every respect are platinum, silver and copper.

In making a denture with aluminum, first obtain a perfect impression. Then make a model of plaster of Paris and marble dust. For a full set, with rubber attachment, adapt base-plate wax to the model exactly as you wish the aluminum plate to be. Make the plate thin, as the metal is strong. Then invest in a two-part flask made for the purpose, much the same as for a rubber piece. After taking it apart to remove the wax, cut grooves from the front and back part of the plate to the outside of the flask. Then lock the flask together again and dry it out. At the same time have the metal heating, and when the cast is dry and the aluminum melted, force the metal into the cast by pneumatic pressure, applied with a rubber bulb. When it is cool, trim the plate and attach the teeth with rubber or celluloid.

When the teeth are to be attached to the aluminum itself, mould the teeth in the flask with the base wax and proceed as before. In fastening teeth in this manner, they should be set slightly apart, on account of a little contraction in the metal.

Dr. Louis Ottofy then read a paper on "Operative Dentistry." Only a partial review of the most important branches of the subject, he said, was possible in the paper. The precious value of time is a matter of consideration for the dentist, even though he may not have an extensive practice. Study each case with a special view to decreasing the amount of labor and shortening the time to the lowest minimum consistent with good work. The practice of inserting extraordinarily large contour fillings is superseded by porcelain settings, or entire porcelain crowns. The former class of work appears to offer, in many cases, service and beauty. Crowns for the anterior teeth are made as simple as possible, with little gold visible. The Logan crown, with improved pin, is, in many cases, the best substitute. They should be ground as little as possible, especially on the palatine or lingual surface, to prevent fracture. Usually the root should be circled by a properly adjusted gold band previous to fitting the crown. The joint is then between the gold band and artificial tooth, and much loss of the oxyphosphate must take place before the root is exposed. For posterior teeth the all-gold crowns are best, and Dr. Patrick's device for making them is worthy of commendation. Those not provided with a proper machine will find the seamless bands and solid gold cusps convenient. Where the occluding teeth are artificial, they may be ground so as to conform to the masticating surface of the crown, but where they are natural, the crown should be shaped with minute care, so as to make it articulate properly with the natural ones. Crowns are preferable to large, unsightly gold fillings for anterior teeth.

The writer has learned nothing new as to the use of gold for filling since last year. Much better and more permanent operations can be made with non-cohesive than with cohesive gold, though the most exposed portion of the filling should be covered with the latter. Margins of cavities are often injured by the pressure necessary to condense cohesive gold, as also by the idea that the cavity must be plugged as tight as possible. Small cavities should be filled entirely with non-cohesive gold, usually by hand pressure. The combination of tin and gold has been tested sufficiently to prove that it has a legitimate place in dentistry. When cavities extend beyond the gum margin to points where it is difficult to insert a filling, this material is especially applicable. It can be finished with burnishers if disks or strips will not reach it. Any discoloration is confined to the material itself. It is not advisable to use it for small fissures in molars and bicuspids, because gold alone can be used to better advantage, and in buccal cavities of molars copper amalgam is preferable. The latter can be introduced more readily, and moisture will not affect it.

Amalgams in general are used more carefully than formerly, and for this reason yield better results. The same attention should be given to the insertion and finishing of an amalgam as to a gold filling, though many of our prominent operators neglect this. The filling should be finished at a subsequent sitting. Copper amalgam is coming into use, and is certainly a good preservative. The principal objection to its use, outside of its discoloration, is that where other metals are inserted in the same mouth patients have complained of a metallic taste, especially when sour food is taken. In one case this was very marked, and the patient spoke of a coppery taste, though not informed that copper had been used. The best use for copper amalgam is in molars, where the cavity extends far below the gum.

The oxyphosphates and gutta-percha are still useful for temporary work. Oxychloride is never used, except when not exposed to the fluids of the mouth. An oxyphosphate will last longer in a mouth, when the saliva is scanty and neutral, than where it is acid or alkaline and viscid.

There is one point in connection with pulpless teeth that deserves attention. Where the tooth has been filled, and from any cause abscess occurs, never resort to the abominable practice of drilling a vent-hole to relieve it. In treating abscesses the use of strong drugs, which, besides destroying the diseased portion, also destroy healthy tissue, should be avoided. One good, thorough application of a suitable drug once a week will yield better results than treating too frequently.

An endeavor should be made to save living pulps in all cases presented under fourteen years. As age advances the chances decrease. Pulps may be dusted with iodoform previous to capping.

The use of matrices in filling saves time and makes it possible to perform better operations. Revolving instruments in the engine enable the operator to work more rapidly, and cause less pain to the patient than the slow-cutting hand instruments.

DISCUSSION.

*Dr. J. W. Wassall*—This is a comprehensive subject. The essayist has necessarily omitted many important points. The suggestion as to the saving of time is a good one. Time is precious to both patient and operator, though the work should not be hurried so fast as to slight it. In filling we should study well to contour our work just to the extent required. There is danger of going to the one extreme of contouring too much, and to the other of not contouring enough. It is also sometimes a nice point to decide between large fillings and crown work. I would prefer, in young patients, to fill, even though it was necessary to contour, and though the filling might last only five or six years. The crown will come soon enough. I am inclined to doubt that the Logan crown is best for anterior teeth. To my mind the regular gold and porcelain crown is preferable to any other for these teeth, and for the posterior teeth the all-gold crown is best.

In regard to bridge-work, I have inserted it in three cases, where it has been found eminently satisfactory, and there is probably a field for it in certain instances, though it is well to sound a note of warning against a too frequent application of the method. The subject of gold is an important one, and should be well discussed. It is the material that we use most in practice. The general rule is to use non-cohesive gold at the cervical margin and along the walls of the cavity, finishing the filling with cohesive. The use of tin and gold dates back forty years, and none can doubt the utility of this material. Soft gold cylinders rolled tight will pack down dense and hard, and may be used to advantage with the matrix. The heavier foils should be used more than they are in finishing fillings. No. 120 will be found very useful for this purpose.

In the use of amalgam I should like to emphasize the fact that the polishing should be done at a subsequent sitting. You will then see the defects if there are any, and can remedy them. Copper amalgam is the latest amalgam hobby in America, and it is possessed of practical value. It is gratifying to look at a good filling of this material in the buccal surface of a third molar.

In the use of cements, there are two secrets of the greatest possible importance. First, use just the exact amount to fill the cavity, so that it will not need trimming. In this way you get the outside gloss of the filling by burnishing, and it leaves a more vitreous surface, which will withstand the action of the saliva better than if cut down. Second, give more time in setting. I usually leave the rubber on an hour after the filling is inserted. If I have other fillings to put in, I tie the rubber around the tooth filled with cement, and proceed with the other work, so that it may be kept dry and set while I am working. If there is no other work to do in the same mouth, I simply tell the patient it is necessary to wait in the office with the rubber on till the filling is perfectly hard, and in the meantime I can work at another patient. Cement fillings treated in this way will last much longer than if inserted in the ordinary manner.

In regard to pulpless teeth with a fistulous opening, the general opinion seems to be that they are the simplest cases for immediate root filling. I must differ from this opinion. When they are filled at once there is sometimes a large chronic opening left at the end of the root, without the operator's knowledge, and the abscess will not heal so long as it is there. It is sometimes necessary, where the end of the root extends up into an opening of this kind, to enter the apical space at the end of the root and cut it off. Where there is a fistulous opening, it is always best to determine whether or not there is an enlarged space at the end of the root before filling. This can be done by passing a broach through the end. If there is sensitiveness when the broach reaches the point that you would judge to be the apex, then there is no cavity; but if it passes on and on, beyond what you must know to be the end of the root, then you may be sure there is a cavity.

It is more necessary to endeavor to save pulps by capping during the formative stage of the tooth than afterward. The process of calcification is not quite complete till adult life, and in patients under twenty it is advisable to make the effort with almost any kind of exposure.

I get a great deal of satisfaction from the use of matrices and separators. For wedging teeth, waxed tape is the most useful material we have. You can procure it in all sizes, and may then saturate it in yellow wax. All teeth will yield slightly when pressed apart, and this material wedges them from the pressure exerted when placed between them, and not from subsequent swelling.

*Dr. Taylor*—Speaking of gutta-percha, we may have a case where the dentist has drilled through the bifurcation of the roots, and by thoroughly drying and filling with gutta-percha we will find that the tissues receive it kindly in these cases. It is also useful for capping nearly exposed pulps, being a better non-conductor and non-irritant than the cements. Another use for it is in deep buccal cavities. Cements sometimes are not thoroughly mixed. Do not burnish an oxyphosphate so as to disturb it while crystallizing, but bring steady pressure to bear upon it.

*Dr. J. N. Crouse*—I have been called upon to speak from the standpoint of how we should treat patients when they come to us for our services. The most important thing is the diagnosis. Not simply to find out how many fillings we have to insert, but how badly the teeth are decayed, and to decide whether the failure is due to neglect on the part of the patient or not. There are several points to be considered when we proceed to put a mouth in order. Our first duty is to instruct the patient as to the proper care of the teeth; especially is this true in the case of children. Then we should consider the pocket-book, so as to judge what class of operations to perform. Not that we should ever do slovenly work, but in cases where the patient cannot afford to pay for expensive work, it may be advisable to use some of the temporary plastics to keep the teeth comfortable and safe till the patient can afford to have more permanent operations performed.

In beginning work for a new patient, take an easy operation at first, till you see what kind of a patient he is going to be, whether he will follow instructions as to cleaning the teeth, and will appreciate good work or not. When patients tell you that fillings are always failing in their teeth, it is more than likely that they themselves are to blame for it. Show your patient the deposits around the teeth and make him appreciate the importance of proper brushing. Tell him what form of tooth-brush to use, and advocate the use of tooth-picks. It should not be considered vulgar, even for ladies, to pick the teeth. Also instruct the patient in the use of floss silk. At the next sitting see what effect your advice has had on the patient. If the teeth are well cleaned and give evidence of close attention, then you may be encouraged to go on and insert good, permanent fillings; but if your instructions have not been followed in any particular, it is well to plainly state to the patient the utter uselessness of placing expensive work in a mouth where so little care is taken of the teeth. The necessity for this kind of instruction holds true, more particularly with children. Get them aroused to the importance of brushing the teeth. I had a boy in the chair one day who told me point blank that he would not brush his teeth. I immediately dismissed him, and sent him home with the remark that I would never work for a boy who refused to take care of his teeth. He appeared quite crestfallen when he saw I was in earnest, and went out of the office reluctantly. I then telephoned his mother what I had done, and told her to give it to him at the other end of the line when he reached home. He came back in a few days with his teeth clean, and to-day I have not a patient who takes more faithful care of the teeth than that boy. This demonstrates the necessity for sharp measures in some instances.

If a patient comes to you with a lot of spaces between the teeth, made by the Arthur method of separating, it is your duty to contour those teeth so that the patient may chew without forcing the food down against the gums and making them sensitive. That is, if the patient will take care of the teeth afterward; if not, it is better to leave the spaces. There is one class of teeth in which separations are especially injurious, namely, those with small necks. A space between these teeth always leaves a pocket at the neck, and food will lodge between the teeth, so that any effort to eat a proper kind of food will result in discomfort. In these cases the patient usually resorts to soaked bread and other soft foods which require little mastication, and which are less beneficial than the solid foods requiring force to masticate them. In contouring these cases the separators come into use. The Parr and Perry separators are both good, but the former is probably the better. The process of wedging is lessened by the use of these appliances, but they must always be used cautiously. It is easier to finish a contour filling now than formerly. We can use disks in the engine, and by directing them with an instrument, may give them any curve we wish. It requires much energy on the part of both operator and patient to go through these operations, but when well performed they will repay it.

In regard to filling materials, if the patient is careless and you cannot arouse him to care of the teeth, it matters little what you fill with, decay will likely recur. Cleanliness is the most important thing for the dentist to attend to. I have used tin and gold in the past, but do not use it so much now. I have had failures through a disintegration of the tin into a powder. I think I can fill nearly as quickly with soft gold cylinders, which I make myself. I roll some tight and others loose, and use the different forms as required. Wedge them in and lap them over the edge of the cavity. Never try to condense the first cylinder thoroughly, but wedge the gold into place. I think there is more impure gold on the market than we suppose. I do not like velvet gold. My experience with it was that it rolled up hard before I could get it well into place against the walls of the cavity. If you want to use a cohesive gold take No. 10, and fold it till you get about eight thicknesses. You can work more rapidly with this than with the heavier foils, say No. 60. Good filling can be made with No. 60, but it takes too long.

Adjourned.

CLINICS AND NEW APPLIANCES.

Two mornings were devoted to this, Wednesday and Thursday.

Dr. T. W. Brophy filled a right upper bicuspid, proximal cavity, demonstrating his loop matrix. Dr. Brophy has made a change in his matrix, whereby one screw can be used with any number of bands, thus lessening the expense.

Dr. J. W. Cormany filled a right central incisor, mesial surface, making a large contour filling with the electric mallet.

Dr. D. B. Freeman demonstrated his various forms of matrices and appliances.

Dr. C. P. Pruyn extracted several teeth after injecting cocaine in the gums as a local anæsthetic. In some of the cases the agent seemed to work favorably, but in one instance the drug produced marked general effects, which necessitated the use of restoratives. Dr. Pruyn stated that this result seldom occurred, but that this case proved the advisability of always being on the alert for such developments.

Dr. C. A. Kitchen inserted a large compound filling in a lower molar, using tin for the cervical margin and bottom of the cavity, and completing with gold.

Dr. W. N. Morrison demonstrated various regulating appliances, among which was the jack screw secured by thin platinum bands. He also replanted a molar that had been extracted in the clinic.

Dr. T. L. Gilmer placed a gold and platinum crown on a lower bicuspid, using the telescope method. A vent-hole was drilled in the grinding surface, and when the crown was in place a small gold wire was driven into the hole and then finished down smooth with the surface of the crown. This does away with the necessity of inserting a gold filling in the vent hole.

Dr. W. H. Taggart exhibited his corundum point and disk maker. This instrument should be in the hands of every dentist who uses corundum points.

Dr. J. G. Reid inserted a tin and gold filling, and Drs. E. D. Swain and K. B. Davis, each a gold filling.

Dr. J. J. R. Patrick demonstrated his method of regulating teeth by means of a large model with movable teeth. His improved appliances for this purpose are as well nigh perfect and as universal in application as it is possible to conceive. The same gentleman also placed a gold crown on a lower bicuspid.

Dr. A. W. Harlan gave a clinic on the treatment of pyorrhœa alveolaris, and exhibited the following new drugs: Ethylate of Sodium, used for destroying fungus growths, in place of chromic acid. It is self-limiting. Oil of Cade is the wood creosote from juniper wood. Iodide Trichloride, one of the newest and best disinfectants, on account of the ease with which it breaks up. It is loosely held together. Guaiaco, one of the principals obtained from wood creosote, to be used as an antiseptic. Benzol, for dissolving gutta-percha. It is more tenacious than the chloro-percha solution. Liquid Vaseline, used as a solvent for hydrochlorate of cocaine, and as a menstruum for antipyrine.

Dr. Fuller, of St. Louis, exhibited a compact case for holding burs and drills for the engine. It may be hung on the wall, and is very readily opened and closed.

A good electrical motor was exhibited by the Belding Motor Co., of Chicago. It is noiseless, simple and cheap.

Dr. H. W. Parsons, of Wamego, Kansas, presented his saliva ejector, warm air injector and atomizer. The working is all accomplished by electricity, and the description of the apparatus is given in a circular, to be had on application.

THURSDAY AFTERNOON.

Dr. Harlan moved that a committee be appointed for memorializing Congress to remove the duty from dental goods coming into this country. Carried.

Dr. W. B. Ames read a paper on "Amalgams." I do not propose, he said, to argue the claims of amalgam to recognition as a filling material. Were I eloquent I might expatiate on its virtues—virtues not born of the material itself, not of its nobility, high character or integrity, but most often of its susceptibility. I am a champion of the plastics. I do not entirely endorse the new departure, yet my conclusions amount to nearly the same as theirs. I would change the wording of their familiar tenet to read, "just in proportion to the difficulties in making fillings that will check and hold in abeyance tooth-destruction, is the use of gold contra-indicated." With this as an axiom, the first question, in a given case is, What is the quality of the tooth structure ? Next, What is the nature of the difficulties to be overcome ? With our available materials this question should be easily answered.

If we had a practical plastic gold it would aid us in the solution of the problem, but so far we have none that can be relied upon. The solution is in the material which combines easy manipulation with permanency. Have we in gold such a material ? Only in certain cases—as in accessible cavities, where plain and easy operations are possible. In bad cavities, where the tooth material would be incompatible with gold (not necessarily electrically), some other material must be made use of. What will it be ? In tin and gold we have a material allied to the plastics. Its adaptation to the tooth-wall is easy, and it is transformed into a homogeneous mass by what I consider primary and secondary galvanic action. But from its nature it is limited in its use, and the disadvantage of color is as marked as in any amalgam. Gutta-percha will not stand attrition, neither will tin foil. Cements may be used as adjuncts, but cannot be relied upon of themselves. Then does not an amalgam easy of manipulation, sufficiently dense, plastic during impaction, and which forms a mechanically perfect stopping, make an ideal filling material, from a thoroughly practical point of view? Have we such an amalgam? From experiments I am satisfied we have.

The "New Departure" gave us many important points, and we have learned little since the publication, ten years ago, of Dr. J. Foster Flagg's work on "Plastics." His conclusions were, that in the heavily tinned amalgams we get bulging or spheroiding, and in the heavily silvered alloys, containing a per cent. of copper, we get very little change of form, even when carelessly manipulated. He put us in the way of judging the qualities of an amalgam from its manipulation.

Cadmium, antimony and zinc, I believe, are of little use in an amalgam. One effect of gold as an ingredient is to render the amalgam very dirty, although an amalgamation of pure gold with mercury will make a clean white substance.

In making tube-tests recently, the conclusions arrived at were, that a good moisture-tight filling can be made by a careful use of many of the higher grades of alloys, and that some of the lower grades may be used beneficially by a certain manner of working them that I have not seen mentioned. In heavily tinned amalgams, the form change is in proportion to the thorough amalgamation; that is, an amalgam just sufficiently mixed to admit of packing changes form less than one made homogeneous by long trituration. This observation strengthened with me a theory that silver and copper in an alloy controlled change of form by becoming only partially amalgamated. The solid particles remaining act as braces to prevent changes incidental to the plasticity of the tin. This theory also explains the crepitation or creaking of silver and copper amalgam. It also accounts for the excellency of copper amalgam.

In this combination of precipitated copper and mercury, I believe that the individuality of the molecules is not destroyed, and we have yet innumerable solid particles of copper with an amalgamated surface, the unamalgamated part acting as a brace against its neighbors to prevent the change of form which would take place in a homogeneous mass. This theory may be unscientific, but I believe that the entire question can be made to harmonize by studying the combining weights, specific gravity and chemical equivalents of the metals. I have not yet formulated my ideas sufficiently to offer them to any advantage.

Amalgamated platinum has not been well understood. I do not refer to what are commonly called platinum alloys, but to amalgamated platinum—platinum and mercury combined. Authors have said that it is difficult of amalgamation, and that its effect in an amalgam is very questionable. Flagg places it last in desirability among the metals. Having produced it myself, I find it has good qualities which heretofore have not been attributed to it. Its only poor quality is that in some combinations it renders a clean amalgam somewhat dirty, and gives it a bad color at the edge. Of itself it remains indefinitely plastic, but when incorporated with other amalgams it generally imparts desirable qualities to the resulting mass, such as toughness and a somewhat leathery consistency. Mixed with sufficient gold to cause hardening, it gives a good amalgam, but one which, for some reason, has not a good surface color. An amalgam of palladium precipitated with platinum sets quickly, according to the amount of palladium. It gets hard and retains the color of pure palladium amalgam, which is a sort of gray, instead of black. With almost any alloy amalgam this material will impart its toughness, and, I think, hasten the setting. The mass becomes hard enough, and the color about the same as the alloy. I use pure copper amalgam in every-day work, and it is in combination with this that I have obtained the best results from platinum, I use it in all proportions. It hastens the setting so that the filling can be trimmed and burnished at the same operation. I sometimes face a copper amalgam with the platinum and copper mixture, thereby getting a good surface that does not turn so black in the mouth as pure copper amalgam.

Palladium amalgam, of itself, is not practicable, on account of its quick setting, but is of value in hardening the surface of other amalgams, and in hastening the setting of the mass. You may harden the surface of an ordinary amalgam filling by rubbing into it, after insertion, small quantities of precipitated palladium. The color is about like that of clean fractured steel or iron.

From very careful experimentation, I believe there is no evaporation of mercury from an amalgam filling, nor do we get any continued effects from chemical action.

### DISCUSSION.

*Dr. Taylor*—All metals in a fluid state have a tendency to assume a globular form. In our amalgams, one of the limitations is due to this. Another limitation is the color; another, lack of edge-strength. These are somewhat modified by recent methods. There is more in the method than in the material. I am opposed to the theory that the lack of amalgamation is beneficial. Take a piece of any alloy, put mercury with it, and it will be drawn into a globule; so if you have an imperfectly mixed amalgam it will assume a globular form. If placed in too dry, you have on the edges a sandy result; if there is too much mercury, you will have a mercurial edge. Amalgam needs thorough trituration; not merely tapping it to draw the mercury to the surface at the edges. It is better to break up the crystallization of amalgam.

Copper amalgam is wonderfully good to keep its form, but there is one objection, that it sometimes will not harden readily. It is not particularly injured by a surplus of mercury, so it is better to have too much than not enough. In the use of copper amalgam we cannot utilize tin for removing the excess of mercury; it will leave a rough and pitted surface to the filling.

The places suitable for the use of copper amalgam are in young persons, in adults with imperfectly organized teeth, and in buccal cavities in second and third molars. A little moisture during its insertion does not materially affect it, but it is best to have it dry. We have not much edge strength with any amalgam, so it is necessary to have good edges to the cavity at nearly right angles.

The supervisor of clinics read his report at this point, and it was discussed in connection with Dr. Ames' and Dr. Ottofy's papers, the discussion of which had been postponed till now.

*Dr. Crouse*—I would like to ask if it is a safe practice to inject cocaine into the gums. It seems to me it would be unpleasant to have a syringe forced into the soft tissues, as we saw it at the clinic. It scared me somewhat to notice how long it took the one patient to rally from its effects. Physicians are growing afraid of it.

*Dr. Sitherwood*—Speaking of matrices, I think the profession has gone wild on this subject. It takes up too much time to apply them. Nearly every operator uses the hand mallet, but I think the automatic or electric plugger will save time and should be used more generally than they are. I deplore so careless a use of cocaine in extracting teeth. There are many physicians lying in their graves to-day owing to this drug, and its use should not be encouraged.

*Dr. Ottofy*—I think the clinics were good. I would criticise, however, the manner in which Dr. Morrison replanted the tooth. It had lain on the table for a time, when it was brought to his notice. He then filled the roots and crown, and when ready to replace it he scraped out the cavity slightly and pushed the tooth back. I question the propriety of such practice. There was no germicide used, and I believe it is safer in these cases to employ some such agent. As to the injection of cocaine in extracting, I was disappointed in the results. I think there was no diminution of pain.

*Dr. Morrison*—A few words as to the case of replanting. I want it distinctly understood that I am afraid of all manner of bugs, but my fear does not extend to the species indicated by Dr. Ottofy. I am careful of the air I breathe, and the water I drink, but I do not believe in the use of such powerful remedies as have been employed in this connection. In my experience I had as good success before these remedies came into use as since, and therefore I feel no fear in my manner of procedure. It is simply a surgical operation, and when we make a wound in any part of the body we do not need to wipe it out with any remedy. All that is necessary is to bring the parts together and allow the serum of the blood to act as a cement. The case at the clinic was not a favorable one. The external plate of the alveolus was somewhat broken and the tissues lacerated in extraction, and although the tooth looked as if it might go easily to place, yet I found some difficulty in returning it.

*Dr. C. N. Johnson*—We must not forget some of the points in connection with Dr. Ottofy's paper. The essayist made the statement that it was never advisable to use tin and gold in fissure cavities in molars and bicuspids, because gold itself could be used to better advantage in these cases. There is one instance that comes to mind, in which I believe tin and gold preferable. In the fissure cavities in children's teeth we can obtain better results by its use than if we attempt the insertion of gold. It can be inserted quicker, and it is seldom necessary to apply the rubber-dam. If it does get slightly wet it is not so much affected as gold, and requires much less consolidation. In regard to the use of the matrix, it seems to me that those gentlemen who condemn it so emphatically have not been in the habit of using it judiciously. For posterior proximal cavities in molars and bicuspids I prefer the Brophy matrix. It is easily adapted and sufficiently yielding at the margins of the cavity to admit of perfect adaptation of the filling material. The argument has been made against the matrix that it consumes time in its application. In reality it requires but a moment to apply it to any ordinary tooth. Another objection has been made that it obstructs the view of the cavity, but with posterior proximal cavities this is not the case. This objection, however, does hold good if the broad band matrix is used in anterior proximal cavities. For these cases I have used a narrow matrix, made by breaking an old watch spring into pieces about half an inch long, and grinding one edge so as to make it convex. The spring has about the proper curve for a matrix, and the convex edge will dip down so as to cover the cervical margin of the cavity, even though it extend below the gum. This matrix is intended to cover only the cervical third of the cavity, thereby forming a pocket at this point, into which the first pieces of gold may be wedged without the necessity of drilling any grooves or pits for starting the filling. It leaves the cavity fully exposed to view, and it can be fastened in position, by means of a wedge dipped in sandarac varnish, in much less time than it would take to form starting points in the tooth structure. One word in regard to the practice of drilling a vent-hole in an abscessed tooth, and leaving it open permanently as a means of relief. It is a painful reflection that it is necessary in this age to condemn such a procedure as that, but it does become necessary, from the fact that it is sometimes practiced yet in localities that are otherwise supposed to be civilized. It is a slovenly, careless, cowardly way of avoiding an issue, and should be criticised in the harshest terms.

The essayist referred to the greater ease with which tooth structure could be cut by the use of revolving instruments in the engine than by those used in the hand. I feel like agreeing with him in this. I am aware that many of our older and better dentists strongly advocate the use of excavators for cutting dentine and shaping cavities, in preference to an extensive use of burs and drills in the engine; but my experience is that with sharp burs, having fine blades, and revolved rapidly, tooth tissue may be cut with greater ease to the operator and less pain to the patient than by any other means. Precision and delicacy of touch is necessary in the manipulation of the hand piece of an engine to obtain the best results, but when this is mastered our work in the preparation of cavities becomes simplified.

*Dr. Crouse*—This subject of the matrix has put a little more talk into me. This appliance is attracting much attention, but I would like to ask if it does not require more time to adjust it than to start the filling without it. You want no retaining points at the cervical margin. If the first piece of gold does not stay in place, hold it firm with an instrument. Do not condense too much in the beginning, but take a large ball of gold and press it into place. I do not like to see a ligature tied around each tooth ; it is too liable to set up periosteal inflammation. I also want to condemn the use of so much tin and gold. It has no advantage over gold alone in fissure cavities in children's teeth. You can use gold in a wet cavity just as well as tin and gold, by wedging it into the fissures. One objection to the matrix is that you cannot contour a filling properly with it, and another is that it is in the way.

*Dr. Pruyn*—The cases in which we use cocaine are varied. In answer to Dr. Ottofy's statement, that there was little diminution of pain from its use, I will say that if he accepts the verdict of the patients, he must believe that there is considerable immunity from pain. In one case it seemed to have no effect, but no drug is uniformly reliable. I am glad the unfavorable case came near the close of the clinic, as it presented a fitting opportunity to extend a warning against using it freely in all cases. It was one of those cases in which we would use no anæsthetic, and I expected trouble. There was general anæmia, and the patient fainted easily on account of having been recently injured. There was no collapse, but if we had pushed the remedy we would have had a sinking.

*Dr. A. W. Freeman*—I fear in some cases we see only its temporary effects. I have heard an eminent gentleman say he would hesitate to use it in the region of the mouth for fear of paralysis of the parts. In regard to filling, one important point is to avoid too much pounding of the gold at the margins, for fear of breaking them.

*Dr. Moody*—I am a country dentist, and wish to speak of Dr. Crouse's extreme advocacy of gold. The fact is, dentists in the country cannot afford to use so much gold ; they cannot get paid sufficiently for it. For an operator to pack gold into teeth so as to make a respectable income out of the fees we get in the country, is simply to pack himself into his grave. I do not wish to lower the status of dentistry, but there are many of our patients to whom it would be a grievous burden to pay a just remuneration for having gold universally used in their teeth. I can show a tin and gold filling in the crown of a tooth that has been there fourteen years. Sometimes it is not so permanent, but properly packed it will often last as long as gold. There are times when it is advisable to use something less trying than gold, and in fissures it can be packed so it will not wear out.

*Dr. Newkirk*—I wish to say something about amalgams. I believe it is not so much in the kind of amalgam as in the way we use it. There is a field for it in the class of cases Dr. Moody has cited, but much depends on its manipulation. When an amalgam is inserted it must remain undisturbed. In large fillings we should hold the material in place till it is firm, and for this purpose I use a matrix made from thin copper, leaving it on till the filling is set. The matrix is made in a moment by having the copper of a suitable width and fastening with soft solder. It is a wonderful assistance in packing amalgam. You can mallet it very dry, and can contour the filling. The matrix may be left on till the following day, and then the filling polished with strips and disks the same as gold. Without the matrix you are not positive that a large amalgam filling is not disturbed before becoming hard. I have some doubt about the copper amalgam since Dr. Ottofy's report of the metallic taste. So much copper is probably not healthy in the mouth. Dr. Ottofy spoke of putting in this filling under moisture. I cannot see the necessity of this in any case. In a child six years old you can hold the rubber on long enough to insert a filling.

To keep buccal cavities in molars dry, I pass the rubber over the tooth and hold it above the edge of the cavity with an instrument in the left hand. I seldom find a cavity I cannot keep dry.

*Dr. Lawrence*—We are better operators than we were twenty years ago, and I would like to ask why it is we are progressing. It seems the spirit which produced this progress has changed. Dr. Pruyn has come as a specialist, and he took this risk for our benefit. It was a practical manifestatiom, and those who saw it were benefited. We have learned more by these demonstrations, and also by illustrations on the blackboard, than by any other means.

Subject passed.

(TO BE CONTINUED.)

